# Promiscuity of enhancer, coding and non-coding transcription functions in ultraconserved elements

**DOI:** 10.1186/1471-2164-11-151

**Published:** 2010-03-04

**Authors:** Danilo Licastro, Vincenzo A Gennarino, Francesca Petrera, Remo Sanges, Sandro Banfi, Elia Stupka

**Affiliations:** 1 CBM scrl - Genomics, Area Science Park, Basovizza, Trieste, Italy; 2 Telethon Institute of Genetics and Medicine (TIGEM), via Pietro Castellino 111, 80131, Napoli, Italy; 3 UCL Cancer Institute, University College London, London, WC1E 6BT, UK; 4 Centre for Gastroenterology, Institute of Cell and Molecular Science, Queen Mary University of London, London, E1 2AT, UK

## Abstract

**Background:**

Ultraconserved elements (UCEs) are highly constrained elements of mammalian genomes, whose functional role has not been completely elucidated yet. Previous studies have shown that some of them act as enhancers in mouse, while some others are expressed in both normal and cancer-derived human tissues. Only one UCE element so far was shown to present these two functions concomitantly, as had been observed in other isolated instances of single, non ultraconserved enhancer elements.

**Results:**

We used a custom microarray to assess the levels of UCE transcription during mouse development and integrated these data with published microarray and next-generation sequencing datasets as well as with newly produced PCR validation experiments. We show that a large fraction of non-exonic UCEs is transcribed across all developmental stages examined from only one DNA strand. Although the nature of these transcripts remains a mistery, our meta-analysis of RNA-Seq datasets indicates that they are unlikely to be short RNAs and that some of them might encode nuclear transcripts. In the majority of cases this function overlaps with the already established enhancer function of these elements during mouse development. Utilizing several next-generation sequencing datasets, we were further able to show that the level of expression observed in non-exonic UCEs is significantly higher than in random regions of the genome and that this is also seen in other regions which act as enhancers.

**Conclusion:**

Our data shows that the concurrent presence of enhancer and transcript function in non-exonic UCE elements is more widespread than previously shown. Moreover through our own experiments as well as the use of next-generation sequencing datasets, we were able to show that the RNAs encoded by non-exonic UCEs are likely to be long RNAs transcribed from only one DNA strand.

## Background

Ultraconserved elements (UCE) have been defined as segments spanning at least 200 base pairs and showing 100% identity between the human, mouse and rat genomes. Further analysis of the distribution of UCEs demonstrates that they tend to be organized in clusters, in regions that are enriched for transcription factors and developmental genes [1]. They have been suggested to be important for functions involving DNA binding, RNA processing and the regulation of transcription and development [2-4], as well as being depleted in regions containing copy number variants [5]. However, our knowledge on these elements is still limited. The mechanisms responsible for maintaining these sequences through evolution are unclear but seem likely to include profound negative selection, suggesting that these segments have important, if not vital, functions [6,7].

Recent studies provide conflicting evidence on their functional role: although it has been shown that many of these elements act as long-range enhancers during mouse development [8], this function is not found for all elements tested and it has been shown that similar proportions of functional enhancers can be found in less constrained sequences [9]. Moreover, deletion of some of these regions in knock-out mice was not associated to any notable phenotype abnormality [10]. These results provided grounds to speculate that UCEs might be simply due to "mutational cold spots", yet it has been shown that these regions are ultraselected [6]. Finally it has also been shown that a larger number of regions in the genome, although shorter, are under similar evolutionary constraints [11].

Recently it has also been shown that some UCEs are expressed and their expression is altered in human tumors, suggesting that these elements may also be involved in cancer development [12]. The transcription of non-coding RNAs from genomic regions acting as enhancers has already been shown to occur in elements with significant sequence conservation, although little is known about the mechanism involved. Indeed the functions of promoter, enhancer and non-coding RNA have been found to overlap in the same DNA fragments with 85-90% mammalian conservation [13] as well as in one UCE [14].

Despite these many findings, the level of constraint observed in UCEs remains as yet unexplained. We decided to further investigate the extent of transcription of UCEs by using an ad-hoc developed microarray as well as several next-generation sequencing datasets. By hybridizing the microarray with total RNA from different mouse embryonic stages and from mouse embryonic stem (ES) cells, and comparing this data with existing next generation sequence (NGS) data, we were able to show that the majority of UCEs which have been shown to act as enhancers during mouse development are also transcribed and investigated salient properties of these transcripts.

## Results and Discussion

### The majority of UCEs are transcribed during mouse development on a single strand

We decided to systematically ascertain to what extent UCEs are expressed and whether the corresponding transcripts can be distinguished from general "transcriptional noise" in the genome. We therefore designed a custom microarray (CustomarrayTM 12K arrays from Combimatrix, Mukilteo, WA) encompassing 3 different probes on each DNA strand of UCEs (of the currently annotated 481 UCEs, probes could be designed for 475), as well as a large number of negative controls (exogenous sequences from bacteria and plants, negative controls used in the Affymetrix platform, rRNAs sequences), which were used to assess the levels of background signal. The sequences of all UCE probes were manually verified to be unique in the genome. This allowed us to assess reliably the levels of expression from both strands of UCE genomic regions during mouse development. In order to define a UCE probe as being expressed, we took into account only probes showing signal intensity above the 90th percentile of the signal distribution in at least two of the three independent hybridizations carried out.

**Figure 1:**
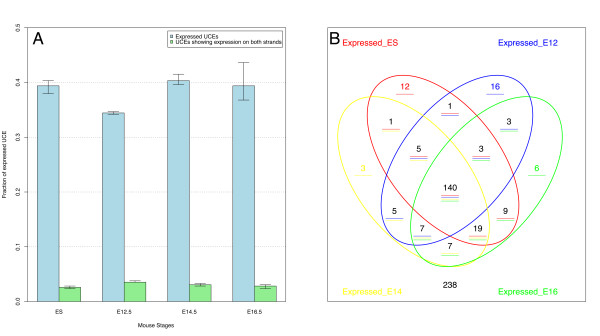
**Custom microarrays analysis**. A) Bar plot of UCEs expressed across 4 mouse developmental stages tested (ES, E12.5, E14.5 and E16.5) based on the analysis of our UCE custom microarray. Blue bars indicate UCEs which show expression on a single strand, green columns indicate UCEs which show expression on both strands B) Venn diagram of UCE transcription results showing the overlap across the 4 stages analyzed. More than half (n = 140, 56%) of the transcribed UCEs are expressed in all the stages analyzed.

The analysis of the microarray data shows that, at each developmental stage, 30 to 40% of the UCEs analyzed are transcribed. However, more than half (56%) of the transcribed UCEs are expressed in at least one of the tested stages (see Figure 1), and 28% of those are found within an exonic location (i.e. overlap the exon of another gene). In total ~50% of transcribed UCEs fall under the exonic category if those considered "possibly exonic" are included (i.e. UCEs overlapping ESTs, mRNAs and gene predictions). Although we have designed probes on both DNA strands of each UCE, only 4% of them display signal on both strands, indicating that most UCEs are parts of transcripts transcribed from only one strand (see Figure 1A). Of these, the majority (59%) is transcribed in all stages tested as well as in ES cells (see Figure 1B). Moreover, when attempting to identify UCEs with a possible differential expression across development, we did not find any UCE showing statistically significant differences in expression between stages (using a corrected p-value cut-off of 0.05). On the contrary, 83% (117/140) of the UCEs transcribed in all stages shows a significantly stable signal across the developmental stages tested.

It should be noted that our experiment can only account for differences across distinct stages of development, while previous experiments accounted only for differences across tissues but not across developmental stages [12]. Thus, if UCEs are regulating transcription in a tissue-specific manner these would not be detected in our study. This data suggests that transcription is a constitutive function in a large fraction of UCEs during development. The only broad difference that was noticeable was the generally lower expression levels in ES cells as compared to other stages, but this was expected since low levels of expression of a broad range of transcripts are known to occur in ES cells.

### RT-PCR validation indicates more widespread transcription

We proceeded to validate the microarray results by carrying out Reverse Transcriptase (RT)-PCR experiments on 31 randomly selected UCEs on total RNA extracted from E14.5 mouse embryos, using 3 independent RNA preparations [Additional file 1]. The RT-PCR analysis was considered positive for expression only for UCEs yielding a specific PCR product validated by sequence analysis in 3 replicates. We used PCRs without reverse transcriptase as a control to ensure that we were detecting a transcribed product rather than a genomic DNA fragment. We observed concordance between the E14.5 microarray data and the RT-PCR results from the same stage in 18/31 UCEs analyzed (12 of which were expressed and 6 of which were not expressed). Among the remaining 13 cases, there are 7 UCEs which were found to be positive only by RT-PCR and no other dataset (i.e. none of the microarray stages, nor in the RNA-Seq data discussed later, except for 2 which are found to be positive in the human microarray dataset), 1 which is found to be negative only by RT-PCR, but confirmed by all other methods, 3 which are not confirmed by the microarray data, but are confirmed by the RNA-Seq data, and 2 which are confirmed by the ES microarray data [Additional file 1, Additional file 2: Supplemental Table S1]. The discordance observed is likely due to several factors. Above all, owing to the fact that the nature and length of UCE transcripts is not yet elucidated, both assays are quite limited. PCRs were conducted with only two pairs of primers per UCE and each UCE was represented with 3 probes per strand. Thus it is likely that testing UCEs with further microarray probes and with further PCR primers would yield different results and greater concordance. Moreover the stringent criteria applied to our microarray analysis is likely to detect a lower number of positives than PCR which is known to be more sensitive. The overall results indicate that our microarray results are likely to under-estimate the number of UCEs that are actually expressed. The RT-PCR validation data would therefore indicate that the number of UCEs transcribed at E14.5 is in the range between approximately 38% (+- 15%, C.I. 95%, as indicated by the microarray data) and 70% (+- 15%, C.I. 95%, which could be extrapolated from the PCR validation).

We wanted to investigate further the potential nature of the transcripts encoded by UCEs and thus performed further RT-PCR analysis for five expressed UCEs (four intergenic and one exonic, UC 475, used as a control) on RNA extracted from either the nucleus or the cytoplasm of mouse ES cells [Additional file 3]. The results indicate that only the control exonic UCE can be detected in the cytoplasmic fraction of RNA, as expected, while the other UCEs tested (intergenic) were found only in the nuclear fraction of ES RNA. Further investigation will be required to ascertain fully the nature of these transcripts, however this data suggests that some intergenic UCEs may act as nuclear transcripts, thus suggesting the possibility that some UCEs might encode ncRNAs. ncRNAs are known to have an extremely wide variety of functions (reviewed in [15]), however given the function of UCEs as enhancers it is tempting to speculate that some of them might act in a manner similar to previously studied highly conserved elements which act as both enhancers as well as ncRNAs [13,14].

### UCE transcription and enhancer function overlap

**Figure 2:**
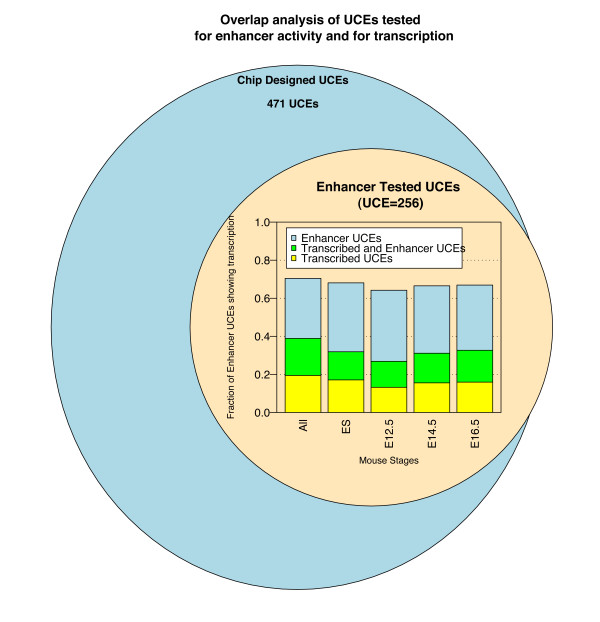
**UCEs transcription and enhancer function overlap**. Overlap between the enhancer dataset (Pennacchio et al, 2006) and the mouse microarray dataset in all samples analyzed, divided by stage. The yellow portion of each bar indicates UCEs that are only transcribed, the green portion UCEs that are transcribed and act as enhancers, the blue portion UCEs that are only transcribed.

Since we performed our expression analysis during mouse embryonic development, we were able to compare our data with previously published data on the function of UCEs as long-range enhancers in mouse embryonic stages [8]. A total of 256 UCEs which were not part of annotated transcripts (intergenic UCEs) were tested for enhancer function and are also present in our microarray design. On this subset of UCEs we were able, therefore, to compare expression and enhancer activity. This comparison showed that 20% of all UCEs tested are transcribed at some developmental stage and act as enhancers during mouse embryonic development, indicating that the two functions can coexist within the same DNA sequence, despite the fact that these regions are intergenic. In the closest embryonic stage analyzed (E12.5) 14% of the tested UCEs still present both enhancer and transcription function (i.e. 27% of those acting as enhancers), indicating that the two functions also can overlap temporally. Some UCEs still present only one of the two functions, i.e. 13% are transcribed but without enhancer activity and 37% behave as enhancers but show no transcription (see Figure 2).

It must be taken into account that the enhancer dataset of UCEs was identified based on experiments carried out at a single developmental stage (E11.5). Therefore, we cannot exclude that the extent of the overlap will be greater when considering enhancer function in other developmental stages. Moreover, due to the different approaches utilized (temporal for our data and spatial for the enhancer dataset [8]) we cannot compare directly expression levels and enhancer function in the same tissue at the same developmental stage. Therefore, we cannot exclude that the enhancer and transcript function might overlap temporally (i.e. same developmental stage) but not spatially (i.e. different tissue) and equally we cannot exclude that a broader overlap could be detected if expression assays had been performed on specific E11.5 tissues in which enhancer function has been observed. A further bias can arise from the construct used for the enhancer assays: since some of them contain two UCE elements within a single construct, it is not always possible to clearly distinguish the contribution of each specific UCE element to the enhancer assay results.

Previous studies have shown that a single ultraconserved element, contained between the *Dlx5 *and *Dlx6 *genes, could act at both DNA and RNA level, and that the ncRNA encoded within them had an effect on the coding transcripts within the locus [14]. Our findings would suggest that the results obtained have a much wider impact than just a few selected elements, since we show that transcription from ultraconserved elements affects more than half of the ultraconserved elements, and also that the enhancer function attributed to some UCEs overlaps in the majority of cases with that of RNA transcript.

### Analysis of the genomic context of UCE elements

**Figure 3:**
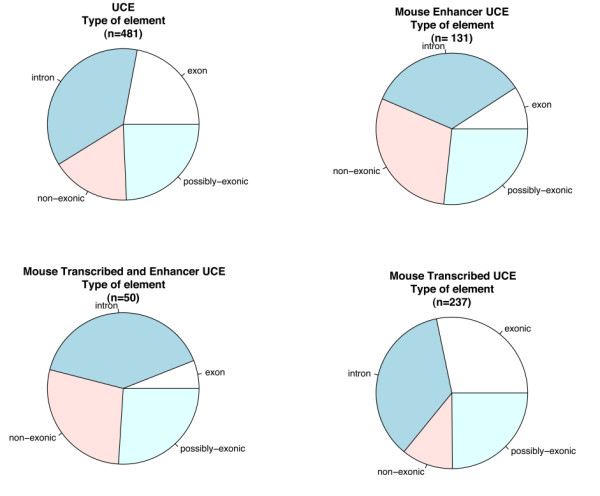
**Analysis of UCEs genomic context**. Classification of UCEs based on genomic context (exonic = overlapping exons of known genes, possibly exonic = overlapping gene predictions, intronic = overlapping introns of known genes, non-exonic = not overlapping any of the above) in comparison with their enhancer function as described by Pennacchio et al, 2006 and transcription function investigated in this study.

Given that UCEs are known to be present in both regions overlapping exons as well as intergenic or intronic regions, we proceeded to verify to what extent the transcription and enhancer functions observed correlate with the genomic context in which they are found. We therefore divided UCEs in the following subgroups: 1) those previously shown to act as enhancers [8] ("Mouse Enhancer" dataset), 2) those shown to be transcribed in our microarray study ("Mouse Transcribed" dataset), and 3) those displaying both of the above features ("Mouse Enhancer and Transcribed"). We then compared these datasets with the genomic context of UCEs as previously defined [1]: a) exonic, for UCEs found within exons of known genes (often referred to also as genic); b) possibly exonic, for UCEs found within portions of the genome for which gene predictions and/or EST evidence indicate the possible correspondence with an exon; c) intronic, for those found within introns and not classified as possibly exonic, and d) non-genic for the remaining UCEs (see Figure 3). Most categories did not show statistically significant enrichment, except for exonic UCEs which are slightly enriched within the transcribed set of UCEs as expected (adjusted p-value 0.0460), and significantly impoverished within the set of UCEs acting as both transcripts and enhancers as well as among those acting as enhancers only (adjusted p-values 0.0137 and 0.0005 respectively). This suggests that UCEs acting as enhancers are more rarely found within exons of known genes (mostly coding genes). Transcribed UCEs, on the other hand, although slightly enriched, as expected, within exons, are present in all UCEs, irrespective of their genomic context: in fact, the largest number of transcribed UCEs in absolute terms is within introns. Taken together these findings suggest that the enhancer function of UCEs negatively correlates with their localization within the exons of protein coding genes despite showing transcription in a significant percentage of cases, indicating that transcripts covering UCEs are very likely to perform different function from traditional coding genes.

Next, we investigated whether transcribed UCEs are preferentially clustered together arguing for a gene-like structure. In order to assess this we verified the distance between all UCEs and compared transcribed UCEs to non-transcribed UCEs. The results indicate quite the contrary: transcribed UCEs are more distant from each other than non-transcribed UCEs at all developmental stages analyzed [Additional file 4]. While it is known that some of the UCEs acting as enhancers are quite close to each other, this result indicates that transcribed UCEs are less likely than non-transcribed UCEs to require proximity to other UCEs for their function. Given the average distances observed among transcribed UCEs (~10 Mbs) they are unlikely to funcion as exons of a gene. We note a bimodal distribution among non-transcribed UCEs with one peak in the 100 kb range, quite distinct from that of transcribed UCEs, and a second peak which aligns well with the distribution of distances among transcribed UCEs. If distances are relevant to the function of UCEs, it would be interesting to verify further whether more distant UCEs which do not appear to be transcribed in our study are perhaps transcribed in other tissues/stages or using other validation means.

Utilizing the genomic context of each UCE element, we also verified whether UCEs, classified according to our expression data and published enhancer data, are found within genes enriched for specific gene ontology (GO) terms. We therefore focused this analysis only on UCEs that have been tested both for enhancer function and for expression during mouse development. The analysis shows that UCEs which act as enhancers (regardless of whether or not they are significantly expressed) are slightly enriched for genes involved in protein binding (p-value 0,02 without multiple test correction). Expressed UCEs, on the other hand, are slightly enriched for GO terms, related to development and regulation of biological processes. We also verified the GO analysis on all UCEs (not only those tested in both studies). This further analysis confirmed the GO terms that had been obtained on the smaller dataset and indicated several additional potential enrichments of UCEs, including an interesting enrichment for UCEs which have only evidence for transcription (and not of enhancer function) to be contained in genes involved in RNA Processing and RNA binding [Additional file 2: Supplemental Table S3 and S4]. Interestingly a similar enrichment for RNA-related functions has recently been reported for UCEs which preserve completely identical sequence across primates and rodents [11]. Given the small size of this dataset, however, it is not possible to draw strong conclusions, and no significant enrichment is found when using multiple testing correction. It could be interesting in the future study to assess transcriptional levels of a larger set of highly conserved elements similar to UCEs [11] and verify whether this GO classification is confirmed.

### RNA-Seq analysis of UCEs indicates significant expression levels, similar to those of other regions acting as enhancers

**Figure 4:**
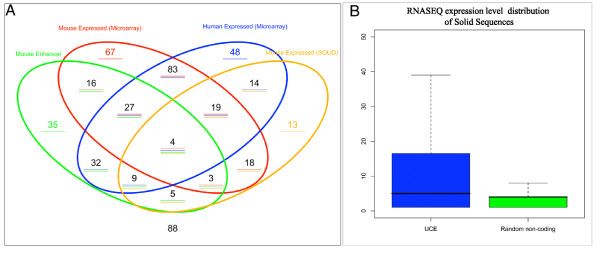
**UCE classification using External datasets**. A) Comparison between the mouse enhancer dataset (Pennacchio et al. 2006) (green oval), our mouse development microarray dataset, (red oval), the human UCE expression dataset (Calin et al. 2007) (blue oval) and the mouse ES cell SOLiD expression dataset (Cloonan et al. 2008) (orange oval). B) Comparison of the SOLiD ES cell RNAseq dataset (Cloonan et al. 2008) for UCEs vs. randomly chosen non-transcribed genomic regions (outliers not shown).

In order to further verify our findings, we also utilized recently published transcription datasets to validate the extent to which UCEs might be transcribed. We re-analyzed two publicly available datasets. First, we used a microarray dataset obtained from the analysis of several human wild-type and cancer tissues [12]. From this study we used the list of UCEs annotated as expressed by the authors. Secondly, we also utilized a recently published SOLiD sequencing dataset obtained from high-throughput RNA sequencing (RNAseq) of mouse embryonic stem cells [16]. The analysis of the RNASeq dataset indicates that UCEs present significantly higher RNA-Seq expression levels as compared to random non-transcribed regions of the genome (Wilcoxon rank sum test p-value 0.009, see Figure 4B), which provides further evidence for the fact that their transcription (shown to be strand-specific in our microarray experiment) is not comparable to general "transcriptional" noise visible across the genome. The comparison with random regions of the genome provided us with a cut-off for bona fide transcribed UCEs based on the RNA-Seq expression levels.

Figure 4A illustrates a complete comparison of the datasets analyzed. The addition of the SOLID dataset (derived from mouse ES cells) indicates that approximately 58% of all UCEs are transcribed in mouse, in line with the lower boundary indicated by the microarray of 40% and the higher boundary indicated by the PCR validations of 70%. Moreover, based on this data, approximately half of the UCEs acting as enhancers show evidence of significant, above noise, expression in mouse in one or more of the datasets utilized. A comparison of this data with data obtained in human tissues allows a further assessment of the phenomenon we investigate on a mammalian scale. The majority of UCEs (62%) that are expressed in human tissues are also found to be expressed in mouse, indicating that the prevalence of this function within UCE regions is broadly conserved across mammals. Notably 32 UCEs which act as enhancers were not detected to be expressed in mouse datasets, but are found to be expressed in human tissues. Although this could be due to different functions in the two organisms, it is likely to be due to the tissue-specific nature of the human dataset, which prompts us to investigate further tissue specificity of UCE expression in future studies. Interestingly, on the other hand, the proportion of UCEs acting as enhancers which overlaps with the human dataset is considerably lower (~30%), indicating that our focus on mouse development has enabled to identify UCE regions which exhibit both enhancer and transcript function more effectively. Only 88 UCEs remain elusive with regards to their potential function after this analysis. Supplementary Table s2 [Additional file 2] summarizes the experimental results obtained for every UCE element using our microarray dataset, previous enhancer screens as well as previous human expression data.

We also verified if we could obtain UCE expression information from a recently published small RNA sequencing dataset obtained from human embryonic stem cells. This experiment utilizes a protocol that filters RNAs based on their length, selecting for transcripts between 18 and 32 nt long [17]. In this dataset, however, we did not observe significant small RNA-Seq expression levels within UCEs, indicating that they are not likely to transcribe small RNAs (data not shown). Further evidence reinforcing this notion is that RNAseq sequences which map to UCEs (filtered for significant expression levels) always cover 100% of the UCE region. Taken together, these observations suggest that the majority of UCEs are not part of short transcripts.

**Figure 5:**
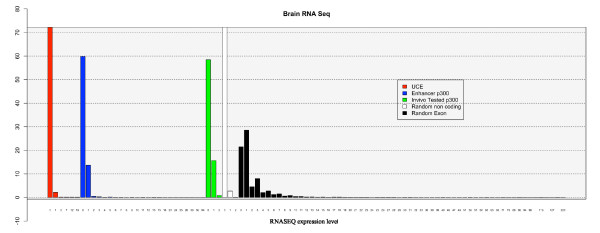
**RNASeq-based expression comparison between random regions, UCEs, p300-bound region and coding exons**. Ultraconserved elements present polyA+ RNASeq based expression levels which are significantly different from random non-coding regions as well as from coding regions, but not from p300 bound regions. Analysis of RNASeq data derived from mouse brain (A. Mortazavi et al, 2008) on several types of genomic regions, i.e. ultraconserved elements (red), randomized set of p300 bound regions (blue, Pennacchio et al, 2009), in vivo tested enhancer regions bound by p300 (green, Pennacchio et al, 2009), random non coding regions in the genome (white) and random coding exons (black).

In order to shed further light on the transcription encoded within UCEs we utilized two recently developed datasets: a recent study in which a genome-wide identification of mouse brain enhancers was performed using p300 ChipSeq [18] as well as a recent study in which RNASeq was performed on several mouse tissues including brain [19]. Using these two datasets, we were able to compare RNASeq- based expression levels in the brain between random regions of the genome, UCEs, p300-bound region likely to encode enhancers, brain enhancers tested in vivo, and coding exons using a Z-score test (see Figure 5). The analysis revealed that UCEs present levels of expression that are very similar to those of in vivo tested enhancers (p-value 0.7), resemble p300-bound regions (p-value 0.012), while they are higher and significantly different from those of random non coding regions of the genome (p-value 1.179978e-25) and lower than and significantly different from those of coding exons (p-value 5.29889e-14).

This data would confirm that the majority of UCEs in which we can detect expression are likely to encode transcripts that are transcribed at significant and, comparable levels with respect to those of other enhancer regions, but lower to those of coding exons. It should be noted, however, that given that our data indicates that UCEs could also encode nuclear transcripts, RNASeq results will be heavily influenced by the RNASeq protocol used and the resulting proportion of nuclear transcripts within the RNA sample sequenced. Given that the data utilized was made using a Poly(A)+ enrichment step, it is likely that the real levels of expression of the nuclear transcripts encoded by UCEs are under-estimated.

In order to further investigate the nature of the transcripts derived from transcribed UCEs we verified the RNA-Seq expression levels of ES cells in the regions proximal to these elements (500 bp upstream and downstream of each UCE). Our analysis indicates that the majority (60%) of UCEs that were found to be expressed in ES cells using the RNA-Seq data also present significant expression levels in the surrounding regions. Half of the UCEs expressed in ES cells present significant levels of transcription on both sides of the element (70% intronic), while 10% present transcription on one side only (80% intronic). The remaining 40% (90% intronic) do not present transcription in nearby regions. In contrast, only 3% of the UCEs found not to be transcribed in ES cells using RNA-Seq presented transcription in the proximal 500 bp regions, of which only 2 UCEs (0.5%) appear to be transcribed both upstream and downstream. Interestingly most of the latter (85%) were exonic, indicating ultra-conserved exons which might be expressed in other stages/tissues, but whose surrounding regions are expressed in ES cells. This result indicates that in the majority of cases the UCE expression investigated in this study might be representative of longer, underlying transcripts which span across and beyond the UCE region. We also inspected the microarray results (since we had designed probes in the surrounding regions) and obtained similar results, i.e. 44% of UCEs found to be transcribed in ES cells present expression in the surrounding regions (56% of UCEs in E12, 42% in E14 and 39% in E16).

Although our data clearly indicates that transcription is an important function of UCE elements, and that this function clearly overlaps with that of enhancer, it remains to be understood to what extent this explains the high evolutionary constrained found in these sequences. The fact that p300-bound regions and in vivo tested enhancers exhibit similar levels of transcription to UCEs would suggest that the co-occurrence of the two functions alone does not explain the constraint. On the other hand, much more in depth investigation of nuclear, tissue-specific transcription should be analyzed before drawing conclusions with regards to their similarity to less constrained enhancers.

It was shown recently that there is a much larger number or regions that are likely to be under similar evolutionary constraint to UCEs, despite their shorter size [11]. Only an in depth analysis of transcriptional levels of a large set of enhancers and conserved regions (highly conserved as well as ultraconserved) performed on nuclear and cytoplasmic RNA is likely to provide further clues with regards to the role of transcription in UCEs.

## Conclusion

In conclusion, we report that the majority of UCEs are expressed throughout mouse development in single-stranded transcripts. Based on our preliminary characterization of these transcripts the RNAs encoded within UCEs are unlikely to be short RNAs and some of them might encode nuclear RNAs. A significant fraction of UCEs appears to act as both enhancers and RNAs during mouse development and RNASeq data indicates that the levels of transcription found in UCEs are likely to be similar to other enhancer regions in the mouse genome. This data provides further evidence and clues with regards to their functional complexity, and reinforces the possibility that the elevated constraint on their sequence might arise from multiple concurrent functions.

## Methods

### RNA Extraction

RNA extraction was performed on mouse embryonic stem (ES) cells, E12.5, E14.5 and E16.5 C57/Bl6 mouse embryos. Mouse embryos were dissected from pregnant females at stages E12.5, E14.5 and E16.5. The dissected embryos were immediately frozen in dry ice and then RNA was isolated using Trizol (Invitrogen) and purified using the RNeasy mini kit (Qiagen). Three independent RNA extractions were performed for each stage and the extracted nucleic acids were treated for DNA contamination with the DNA-free Kit (Quiagen) to prevent DNA carryovers. The cytoplasmic fraction of RNA from ES cells was extracted according to the Maniatis protocol. The quality and concentration of the RNA was determined by measuring the absorbance at 260 and 280 nm using the Nanodrop nd-1000 Spectrophotometer while the RNA integrity was determined using the Agilent Bioanalyzer RNA 6000 Nano chip according to manufacturer instructions.

### ES Cell Culture

The feeder-independent mouse Embryonic Stem (ES) E14 Tg2A.4 cell lines were grown on gelatin (0.1%), plastic-coated petri dishes in Dulbecco's modified Eagle medium (DMEM, Invitrogen) supplemented with 15% (vol/vol) heat-inactivated Fetal Bovine Serum (FBS, Euroclone), 2 mM L-glutamine, 1 mM MEM sodium pyruvate, 0.1 mM 2- mercaptoethanol and 1,000 units/ml leukemia inhibitor factor at 37°C in a humidified chamber supplemented with 5% CO2.

### Reverse Transcriptase RT PCR

Reverse Transcriptase (RT)-PCR was performed in triplicate on total RNA extracted from E14.5 mouse embryos. The UCEs were chosen in a different laboratory from the one performing the RT-PCR experiments, without communicating the result expected on the basis of the microarray data. To prepare cDNA synthesis, we used the Quantitect Reverse Transcription kit (Qiagen, Inc.) starting from 1 μg of DNAse-treated RNA. To further exclude genomic DNA contamination, RT- (no reverse transcriptase added) reactions were used as negative controls. Oligonucleotide sequences and PCR conditions are available upon request. Only RT-PCRs, which were positive in all three replicas, negative in the RT- and confirmed by sequencing analysis were considered as positive.

### Custom Microarray Design

The electrochemically synthesized oligonucleotide microarrays used in this study are CustomarrayTM 12K arrays (Combimatrix, Mukilteo, WA). The oligonucleotide sequences and arrays were designed using as a reference the murine genome sequence from the Ensembl release 46. The arrays consist of ~12,000 oligonucleotide probes. For each UCE, three probes were designed, on both DNA strands of the UCE element. We mapped the published UCE sequence to the unmasked murine genome and, through the use of bioperl scripts, we also retrieved the sequences 500 bp upstream and downstream of the mapped UCE and designed probes in these regions. Moreover if the UCE mapped inside known exons, we designed probes also for the overlapping exon while if the UCE mapped between exons, we designed probes for the nearest upstream and downstream exons. The probes have been further selected according to their melting temperature using the Combimatrix ProbeWeaverTM Software. The dataset of all UCEs analyzed, their sequences and the probes used is available at http://biodev.cbm.fvg.it/cgi-bin/uce/total_uce.pl. The microarray design and data has been deposited to the GEO database, as dataset GSE19371.

### Microarray Labeling and hybridization

Biotin-labeled cRNAs was produced with Illumina Total Prep RNA Amplification Kit (Ambion), according to manufacturer protocol, starting from extracted RNA. The arrays were enclosed within the supplied hybridization chambers, filled with H2O and incubated in a rotisserie hybridization oven for 10 min at 65°C to remove adsorbed oxygen from the slides. The arrays were then incubated at 45°C for 30 min in prehybridization solution (6× SSPE (Ambion), 0.05% Tween-20 (Sigma Aldrich, St. Louis, MO), 20 mM EDTA (Ambion), 5× Denhardt's solution (Sigma Aldrich), 100 ng/μl salmon sperm DNA (Sigma Aldrich), 0.05% SDS (Sigma Aldrich)). Biotin-labeled RNA was added to the hybridization buffer (100 μl total volume, 6! SSPE, 0.05% Tween-20, 20 mM EDTA, 25% formamide, 100 ng/μl salmon sperm DNA, 0.05% SDS) heated at 95°C for 3 min, chilled briefly on ice, added to microarray slides, and incubated at 45°C overnight. Slides were washed for five successive washes of 5 min each in Wash I (6! SSPE, 0.05% Tween-20), Wash II (3× SSPE, 0.05% Tween-20), Wash III (0.5× SSPE, 0.05% Tween-20), Wash IV (2× PBS, 0.1% Tween-20), and Wash V (2× PBS (Sigma Aldrich). Wash I was performed at 45°C with all subsequent washes at room temperature. The slide was removed from the hybridization chamber then immediately stained with HRP using biotin-avidin chemistry according to manufacture protocol and read using a ElectraSenseTM Reader using the revelation solution

### Microarray Analysis

The raw data signal was exported from the ElectraSense Analysis Software, loaded into the R/Bioconductor program [20], thanks to the Biobase package, and finally converted into a Bioconductor Expressionset Object. Gene expression data were normalized by Quantile regression [21]. Probe signal was considered positive if present in at least two of three independent hybridizations above the 90th percentile of the signal distribution. We also required for the signal to be higher than the average of the negative probe signal + 2 standard deviations, which was the case for all probes selected, given the 90th percentile cut-off applied The Limma package [22] was used to perform the statistical analysis to identify differentially expressed UCEs. A cut off after the adjustment of P-values to control the false discovery rate (FDR) [23] was set to p < 0.05 as set out by the MAQC consortium guidelines [24]. In order to identify constitutively expressed UCEs (i.e. with stable signal across development) we used a method previously developed by our lab [25]. Briefly this involved calculating the MFC (maximal fold change), i.e. the ratio of the maximum and minimum expression values for all microarrays performed in the experiment, and the CV, i.e. the coefficient of variation. We considered as constitutively expressed only those UCEs having: MFC < 2, CV < 0.05, an average signal intensity larger than the 5th percentile of the distribution of the signals (to avoid the classification of UCEs with very low signals) and a signal intensity larger than two standard deviations from the mean of the negative probes. For the Gene Ontology (GO) analysis we performed two analyses: one [Additional file 2: Supplemental Table S4] which focused only on UCEs that have been tested for both Enhancer and Transcription activity, another [Additional file 2] which analyzed GO context of all UCEs, regardless of whether they had been tested for enhancer and/or transcription activity. The analysis has been performed within the Bioconductor environment using the packages GO.db version 2.2.0 and GOstats [26] version 2.6.0 with the hyperGTest function.

### Selection of random non-transcribed region

In order to obtain a comparable dataset of random non-transcribed regions of the genome for each UCE analyzed, we selected randomly 3 regions of the same length with no evidence of transcription and/or presence of repeated sequences. We exploited the annotation of the Ensembl database (mouse genome, version 49) and selected regions presenting no 'dna align features' (i.e. no alignment with known cDNAs, ESTs, etc) with a score higher than 75, no 'protein align features', no 'prediction transcripts' and no 'repeat features' overlapping them. Finally we only selected regions that did not contain 'N' nucleotides.

### UCE classification using External datasets

We classified UCEs as "exonic", i.e. if they overlapped known exons, "intronic", if they fell within introns of known genes, "possibly exonic" if they overlapped regions which present some evidence of genes (e.g. gene predictions, EST alignments, etc) and "non exonic" if they did not fall in any of the above. A single nucleotide of overlap was considered sufficient for UCEs to be classified in the above categories. We then compared these annotation categories among 4 datasets: a "UCE" dataset, containing all UCEs; a "Mouse Transcribed UCE" dataset, containing UCEs which were found to be expressed during mouse development using our custom microarray experiment, a "Mouse Enhancer UCE" dataset, containing UCEs which have been shown to act as enhancers [8], and a "Mouse Transcribed and Enhancer" dataset, containing UCEs which were found to act as enhancers previously [8] as well as found to be expressed during mouse development in our experiment. On each of these sets we performed a Perfect test followed by Hochberg correction to determine if the proportions of annotation categories deviated significantly from the proportions found in the complete UCE dataset. The dataset of UCEs tested for enhancer activity was downloaded from the webpage http://enhancer.lbl.gov/ and has been mapped to UCEs sequences using wblast tool and Perl parsing scripts. The dataset of UCEs expressed above background levels in 19 human normal tissues [12] was downloaded from the webpage of the manuscript. Expressed UCEs were selected as annotated by the authors (i.e., those displaying a signal higher than the average of blank spots on the array + 2 standard deviations). The RNA-Seq dataset obtained from mouse embryonic stem cell (using SOLiD sequencing) was downloaded from the Supplemental Data of the manuscript [16], using the "ES junction" BED track. Forebrain, Midbrain and Limb p300 Peak bound regions were downloaded from the Supplementary Data of the manuscript [18] and filtered for FDR lower than 0.01 as suggested in the manuscript. Solexa RNA-seq normalized wigglegrams of unique reads from adult brain, liver and skeletal tissues were downloaded from the Supplementary Methods of the manuscript [19]. Reads from the above experiments were mapped to UCE regions, randomly chosen mouse exons (mouse genome, version 49), random non-transcribed regions, and p300 bound regions using a custom R script. The comparison of RNASeq- based expression levels in the brain between random regions of the genome, UCEs, p300-bound regions likely to encode enhancers, brain enhancers tested in vivo, and coding exons has been performed using a Z test with 10,000 random sampling.

## Competing interests

The authors declare that they have no competing interests.

## Authors' contributions

DL carried out the Custom Microarray Design, Microarray Labeling and hybridization, statistical analysis and the UCE classification using External datasets. VAG carried out the RNA Extraction, ES Cell Culture and Reverse Transcriptase RT-PCR analysis. FP participated in the Microarray preparation and RNA quality controls. RS carried out the Selection of random non-transcribed region. DL and VAG drafted the manuscript. ES and SB conceived and co-ordinated the study and wrote the final version of the manuscript. All authors read and approved the final manuscript.

## Supplementary Material

Additional file 1 **Supplementary Figure 1**. The summary of results for PCR tested UCEs. Click here for file

Additional file 2 **Supplementary Tables**. Table 1,2 The summary expression results for each UCE. Table 3,4 Tables summarizing the Gene Ontology Analysis. Click here for file

Additional file 3 **Supplementary Figure 2**. RT-PCR analysis for five expressed UCEs on RNA extracted from either the nucleus or the cytoplasm of mouse ES cells. Click here for file

Additional file 4 **Supplementary Figure 3**. The analysis of distance between UCEs Click here for file
