# Supplementary figures and images for: Promiscuity of enhancer, coding and non-coding transcription functions in ultraconserved elements
Source: BMC Genomics. 2010 Mar 4;11:151. doi: 10.1186/1471-2164-11-151 (PMC2847969; doi:10.1186/1471-2164-11-151)

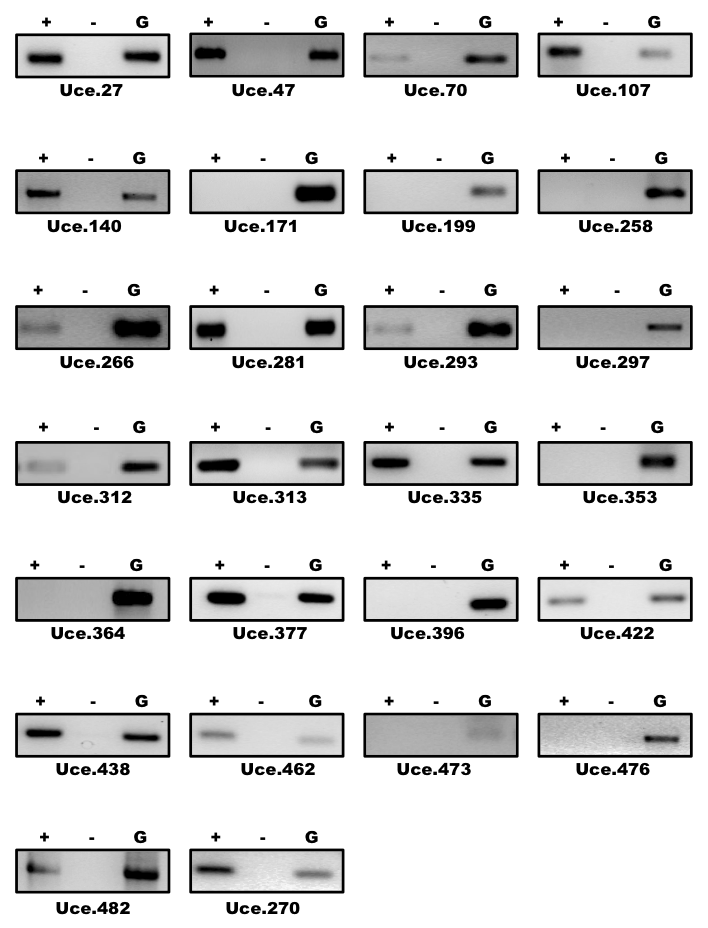

Supplement: Additional file 1 — Supplementary Figure 1. The summary of results for PCR tested UCEs. [file 1471-2164-11-151-S1.TIFF]

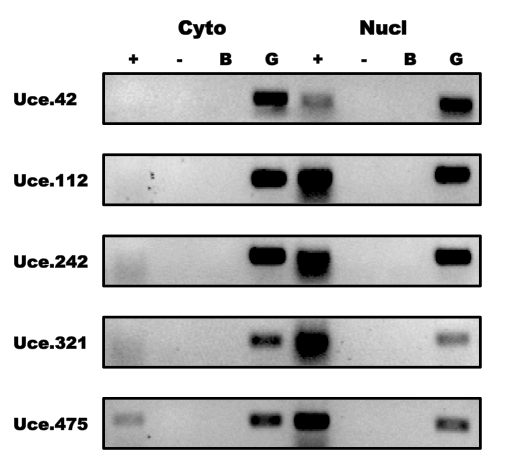

Supplement: Additional file 3 — Supplementary Figure 2. RT-PCR analysis for five expressed UCEs on RNA extracted from either the nucleus or the cytoplasm of mouse ES cells. [file 1471-2164-11-151-S3.TIFF]

# UCE ES

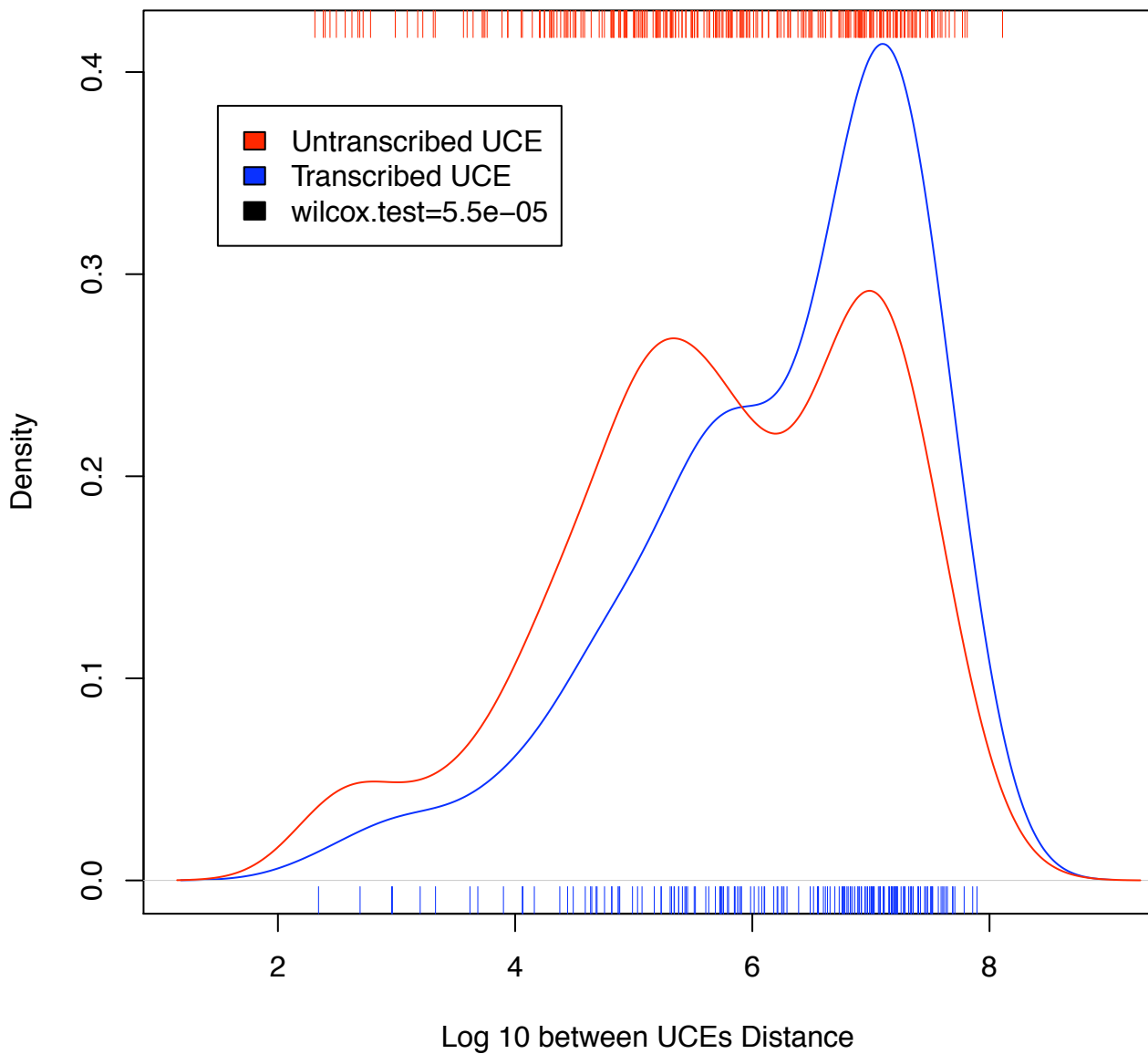

# UCE E12

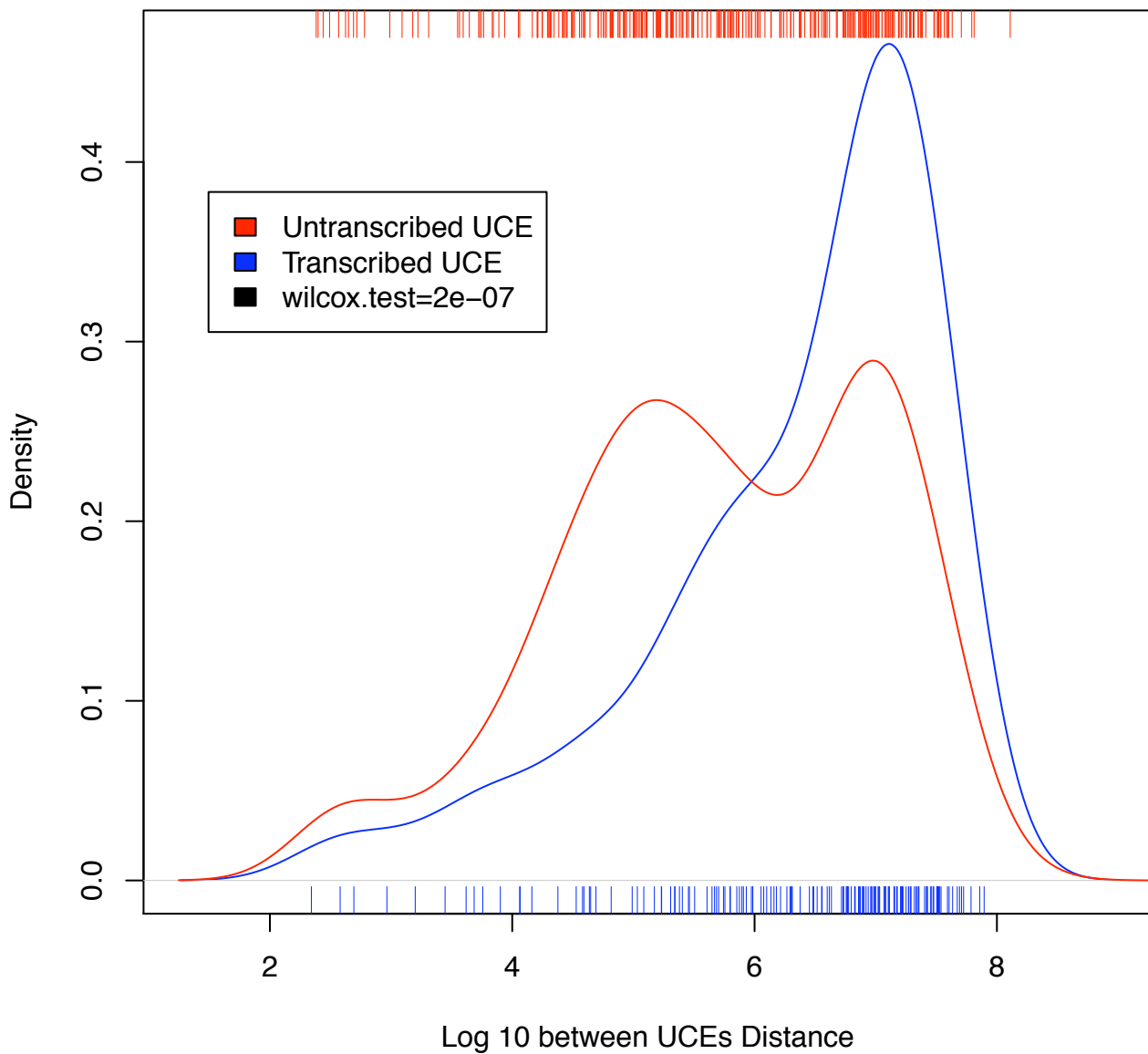

# UCE E14

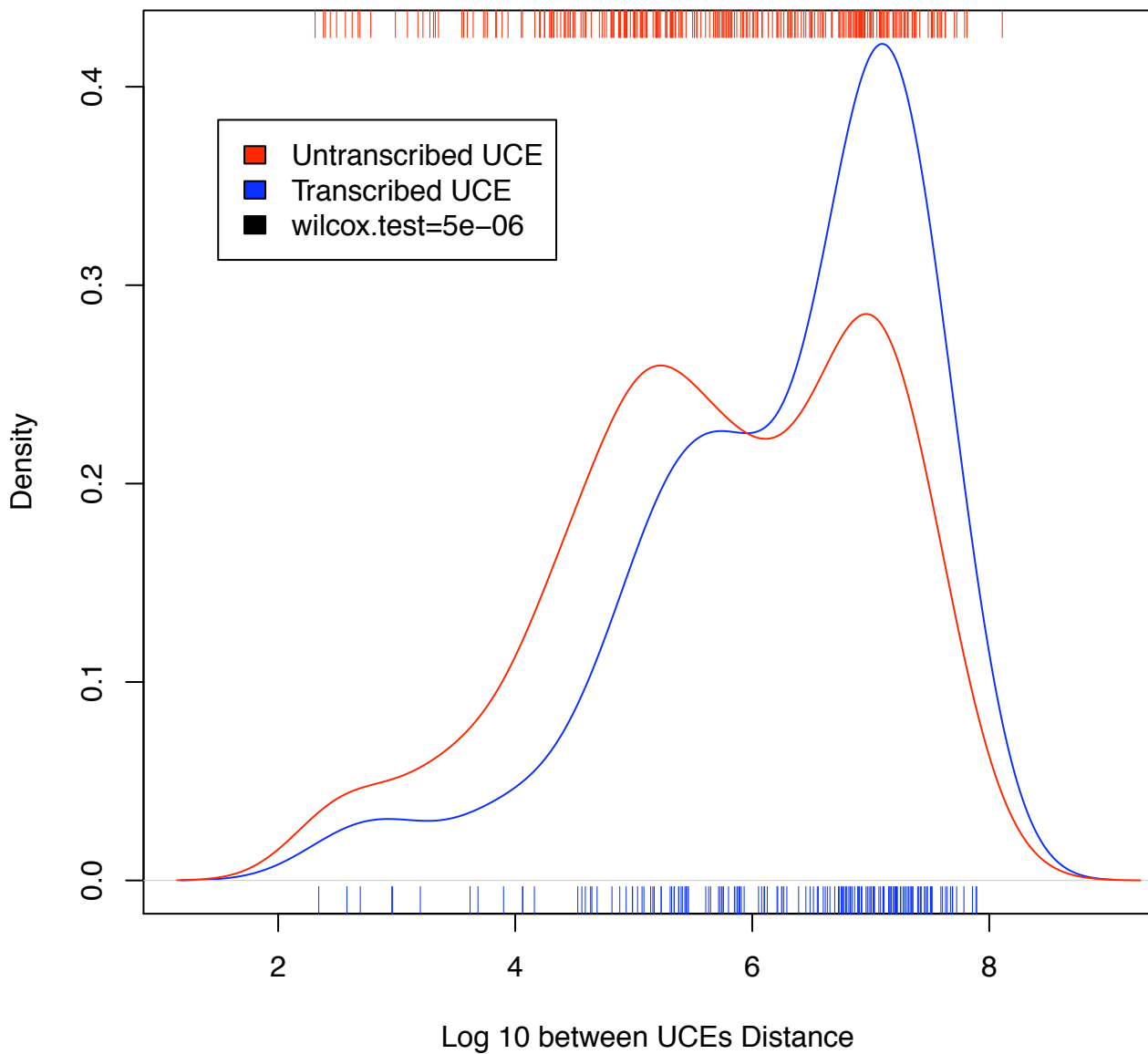

# UCE E16

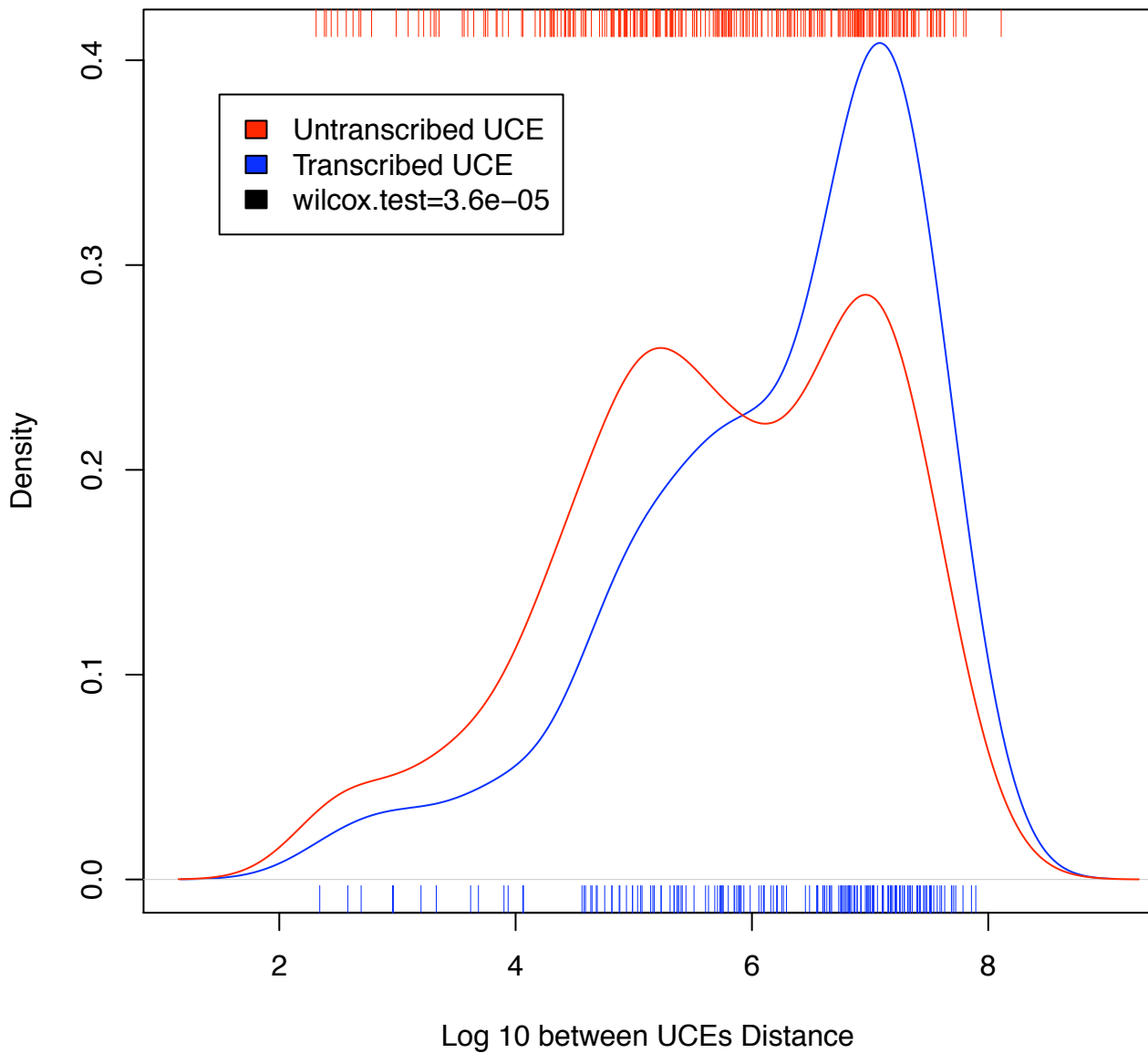

Supplement: Additional file 4 — Supplementary Figure 3. The analysis of distance between UCEs [file 1471-2164-11-151-S4.PDF]
